# Predicting the prognosis of patients with sudden sensorineural hearing loss by analyzing the audiometric curve of the unaffected ear

**DOI:** 10.3389/fneur.2025.1575122

**Published:** 2025-05-30

**Authors:** Xuan Sun, Yahan Zhao, Yi Li

**Affiliations:** Beijing Tongren Hospital, Capital Medical University, Beijing, China

**Keywords:** sudden sensorineural hearing loss, pure-tone audiometric threshold, cluster analysis, prognosis, unaffected ear

## Abstract

**Objective:**

This study aims to extract potential information from the audiograms of the unaffected ear in patients with unilateral sudden sensorineural hearing loss (USSNHL). It explores the relationship between the characteristics of the audiograms of the unaffected ear and the treatment effectiveness for USSNHL. Additionally, the research presents the findings in a way that enhances communication and allows for verification.

**Methods:**

The study employs piecewise curve fitting to simplify the changing trend of audiograms in the unaffected ear of USSNHL patients into the slopes of three straight lines. Utilizing Python, the research team conducts a cluster analysis on the 229 patients’ audiometric characteristics and trains the clustering results into an algorithm model. After clustering, the team applies statistical methods such as regression analysis to explore the correlation between the clustering results and the therapeutic efficacy.

**Results:**

The study completes the clustering analysis and encapsulates the trained model into an executable program. The algorithm clusters the patients into Cluster X and Cluster Y based on the audiogram characteristics of the unaffected ear. The clustering results demonstrate a significant correlation with the treatment efficacy. Regression analysis shows that Cluster Y patients achieve an average improvement in hearing threshold post-treatment that is 6.52 dB higher than that of Cluster X. The relative risk of “No improvement” for Cluster Y is half that of Cluster X. Additionally, age and the audiogram type of the affected ear also contribute to the prognosis of USSNHL to varying degrees. Furthermore, the research team submits the trained clustering model and corresponding spreadsheet as attachments, facilitating dissemination and validation.

**Conclusion:**

Regression analysis confirms that the clustering results are independent factors indicative of the prognosis in patients with USSNHL. The data exerting the most significant influence on clustering analysis outcomes were derived from the evolving auditory threshold patterns in the posterior segment of audiometric curves obtained from unaffected ears. This observation indicates a strong correlation between mid-to-high frequency threshold progression in the contralateral ear and clinical prognosis among patients with USSNHL. The clustering methodology demonstrated robust classification efficacy for auditory data lacking explicit cutoff values, ultimately enabling refined patient stratification through multidimensional pattern recognition.

## 1 Introduction

In the treatment guidelines for sudden sensorineural hearing loss (SSNHL) in the United States, SSNHL is defined as a subset of sudden hearing loss that is sensorineural in nature, occurs within a 72-h window, and consists of a decrease in hearing of 30 dB affecting at least three consecutive frequencies (1). Due to the unclear etiology of SSNHL, the prediction of its prognosis remains challenging, thereby complicating communication between clinicians and patients. An increasing number of studies focus on exploring specific indicators to predict the prognosis of SSNHL, such as hematological indicators (2), immunological indicators (3, 4), and imaging indicators (5). The aim of these studies was two-fold: firstly, to establish effective clinician-patient communication regarding disease progression and facilitate clinical evaluation of severity stratification, thereby enabling formulation of initial therapeutic protocols or implementation of necessary modifications such as medication dosage escalation or adoption of combination therapy regimens. Secondly, prognostic predictors associated with SSNHL frequently manifest as biomarkers indicative of potential underlying etiopathogenesis or pathophysiological mechanisms inherent to this disorder. Therefore, investigating the influencing factors of SSNHL prognosis can provide novel insights into exploring its etiology and pathological mechanisms.

The hearing of the unaffected ear may reflect the pre-morbid auditory status of patients with USSNHL, thereby offering insights into the functional state of the cochlea prior to the onset of the condition. This possibility is posited to exert an influence on the prognosis of SSNHL. The view that the hearing status of the unaffected ear may influence the prognosis of SSNHL was proposed long ago (6). However, no studies have yet clarified how to appropriately evaluate the hearing status of the unaffected ear, nor have they determined the extent to which different hearing statuses of the unaffected ear affect clinical outcomes. The limited research on the unaffected ear may be attributed to the lack of appropriate tools for extracting auditory information from the unaffected ear and effectively categorizing patients based on this data.

**Figure 1:**
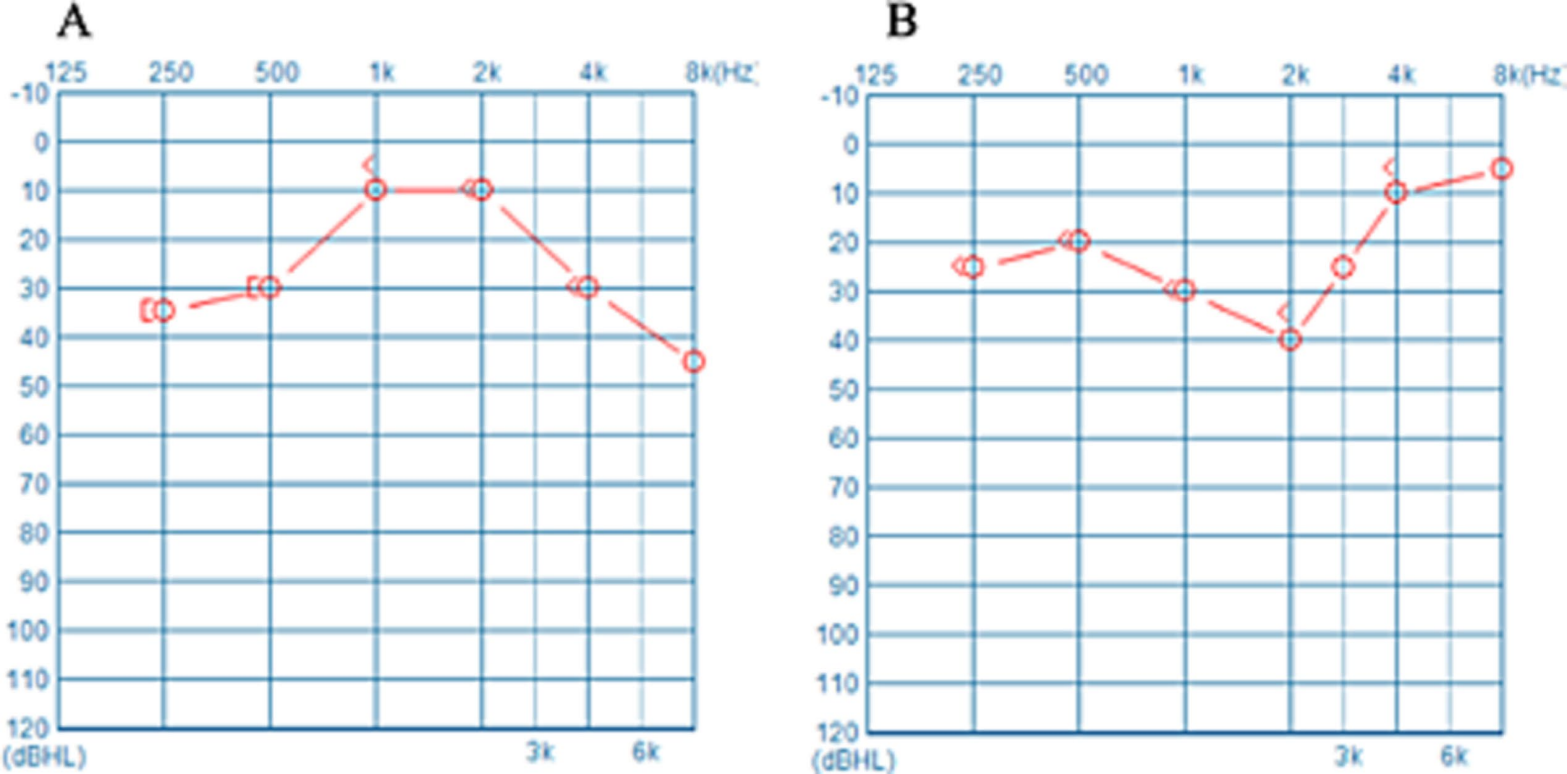
**(A,B)** Represent the PTA thresholds of the unaffected ear of two USSNHL patients at our institution. Despite the comparable average hearing thresholds across the spectra, marked disparities in the pattern of the curves emerge at their respective extremities.

Few studies have corroborated the notion that dysfunction in the average auditory threshold of the unaffected ear influences the prognosis of patients with USSNHL. According to the distribution function of human auditory frequencies on the basilar membrane, the commonly used pure-tone audiometry (PTA) frequencies, ranging from 250 Hz to 8,000 Hz, primarily reflect the hearing status of approximately 60% of the cochlea’s central segment (7). Consequently, the functionality corresponding to 40% of the basilar membrane’s length remains unrepresented. We posit that the trend of threshold curve changes at the margins can serve as a clue to the unrepresented functionality of the 40% length of the cochlea, thereby enabling a more comprehensive estimate of the overall cochlear function. Figure 1 depicts the PTA curves of the unaffected ear in two patients with USSNHL. The average threshold levels of the unaffected ear in both patients are similar, yet the trends at the extremities of the threshold curves differ significantly. The designation of “unaffected ear” is determined by comprehensive evaluation of patient history and chief complaints, specifically defined as the contralateral ear in patients presenting with USSNHL as their primary concern, without reported involvement of the contralateral ear during the acute episode and with explicit denial of prior sudden deafness history in that ear. Furthermore, it is crucial to clarify that the term “unaffected” does not imply otological normality; rather, this operational definition acknowledges potential subclinical audiological variations, which constitutes the fundamental rationale for implementing systematic audiological profiling and classification of threshold characteristics in these ears. Curve A shows a marked downward trend at both high and low frequencies, whereas Curve B shows an upward trend at the extremes. This phenomenon may indicate that Patient A performs poorly at hearing frequencies not represented in the audiogram. We propose the following hypothesis: despite the average pure tone hearing thresholds being similar in the audiogram, Patient A may exhibit poorer cochlear function, resulting in a worse prognosis of USSNHL. It is an unexplored hearing feature in the unaffected ear.

In this study, we initially embraced the diagnostic criteria for SSNHL in China, collecting audiograms from the unaffected ears of USSNHL patients for cluster analysis. The clustering algorithm categorizes the USSNHL patients into two clusters based on the proximity of characteristic data within the audiograms, thereby uncovering the hidden associations between the data (8). After preliminarily validating the differences in treatment efficacy between the two patient clusters, the clustering algorithm was trained as a predictive model to ensure its general applicability, communicability, and verifiability. Subsequently, we reimplemented internationally recognized diagnostic criteria to exclude patients failing to meet international standards, thereby establishing a refined patient cohort. Internal validation of the model was conducted using this rigorously defined population, ensuring model generalizability across varying diagnostic frameworks. Ultimately, through regression analytical approaches, we quantitatively delineated the therapeutic efficacy-clustering outcome relationship and identified pivotal data determinants influencing cluster differentiation.

## 2 Materials and methods

### 2.1 Handling of research subjects

#### 2.1.1 Selection of research subjects

As per Chinese guidelines, SSNHL is defined as a subset of sudden hearing loss that is sensorineural in nature, occurs within a 72-h window, and consists of a decrease in hearing of 20 dB affecting at least two consecutive frequencies (9). This study collected data from 250 patients diagnosed with SSNHL and treated at our hospital from August 2022 to April 2023. The diagnostic criteria employed are those stipulated in the “Guidelines for the Diagnosis and Treatment of Sudden Hearing Loss (2015)” (hereinafter referred to as the “Chinese Guidelines”) mentioned in the preceding text. Excluded were: ① patients with bilateral onset and patients with prior history of contralateral SSNHL; ② patients whose time from onset to consultation exceeded 7 days; ③ patients without detailed treatment records; ④ patients subsequently diagnosed with specific diseases such as vestibular aqueduct enlargement or Meniere’s disease during follow-up treatment; ⑤ patients who refused or were deemed unsuitable for standardized treatment according to “Chinese Guidelines” due to the presence of other conditions (such as poorly controlled hypertension, diabetes, or hemorrhagic disorders). A total of 229 patients were initially included, and the auditory information from their unaffected ears was utilized to train the clustering analysis model and to validate the clustering results preliminarily. Subsequently, we excluded an additional 52 individuals using the above international diagnostic criteria. We utilized the data from the remaining 177 individuals to conduct model revalidation and regression analysis. In addition to the clustering results mentioned above, the data incorporated into the regression analysis included other factors widely recognized by the public as influencing the prognosis of USSNHL patients, such as age and the type of audiogram for the affected ear. Conventional baseline data, including gender and the side of onset, were also included in the regression analysis.

#### 2.1.2 Therapeutic strategies

All enrolled patients were classified into hearing loss types according to the affected ear’s PTA thresholds in the early stages of onset, following the “Chinese Guidelines.” The classifications based on the audiogram of the affected ear included “upsloping,” “downsloping,” “flat,” and “profound.” Treatment recommendations based on the specific type of hearing loss were administered in the otolaryngology emergency department of our institution within the first 14 days. For the “upsloping” and “downsloping” hearing loss types, combined treatment with glucocorticoids and *Ginkgo biloba* extract was utilized; for the “flat” and “profound” hearing loss types, a combination of glucocorticoids, *Ginkgo biloba*, and baclofen was employed. Treatment may be concluded prematurely if the patient’s hearing in the affected ear returns to baseline levels within 14 days.

#### 2.1.3 Criteria for efficacy assessment

Fourteen days post-treatment, investigators re-evaluated the PTA thresholds and assessed efficacy based on Siegel’s criteria (10), with the evaluation standards outlined in Supplementary material. “Complete recovery,” “Partial recovery,” and “Slight improvement” were all classified as indications of “Improvement.” In cases where conflicting assessments emerged during hearing outcome evaluation using Siegel’s criteria, the inferior outcome classification was systematically adopted to ensure conservative evaluation. This resolution protocol prioritized clinical prudence over optimistic interpretations when confronting ambiguous audiometric progression patterns. We employed three metrics to assess the effect of the treatment. The first metric is the improvement in hearing threshold, calculated as the average hearing threshold (250 Hz–8,000 Hz) of the affected ear before treatment minus the average hearing threshold of the affected ear after treatment. The second metric was the cure rate of treatment, determined by the proportion of cases experiencing “Complete recovery” within the population. The third metric was the improvement rate of the treatment, which assessed the proportion of individuals showing any improvement (“Complete recovery,” “Partial recovery,” and “Slight improvement”) relative to the total population.

### 2.2 Construction of clustering model

**Figure 2:**
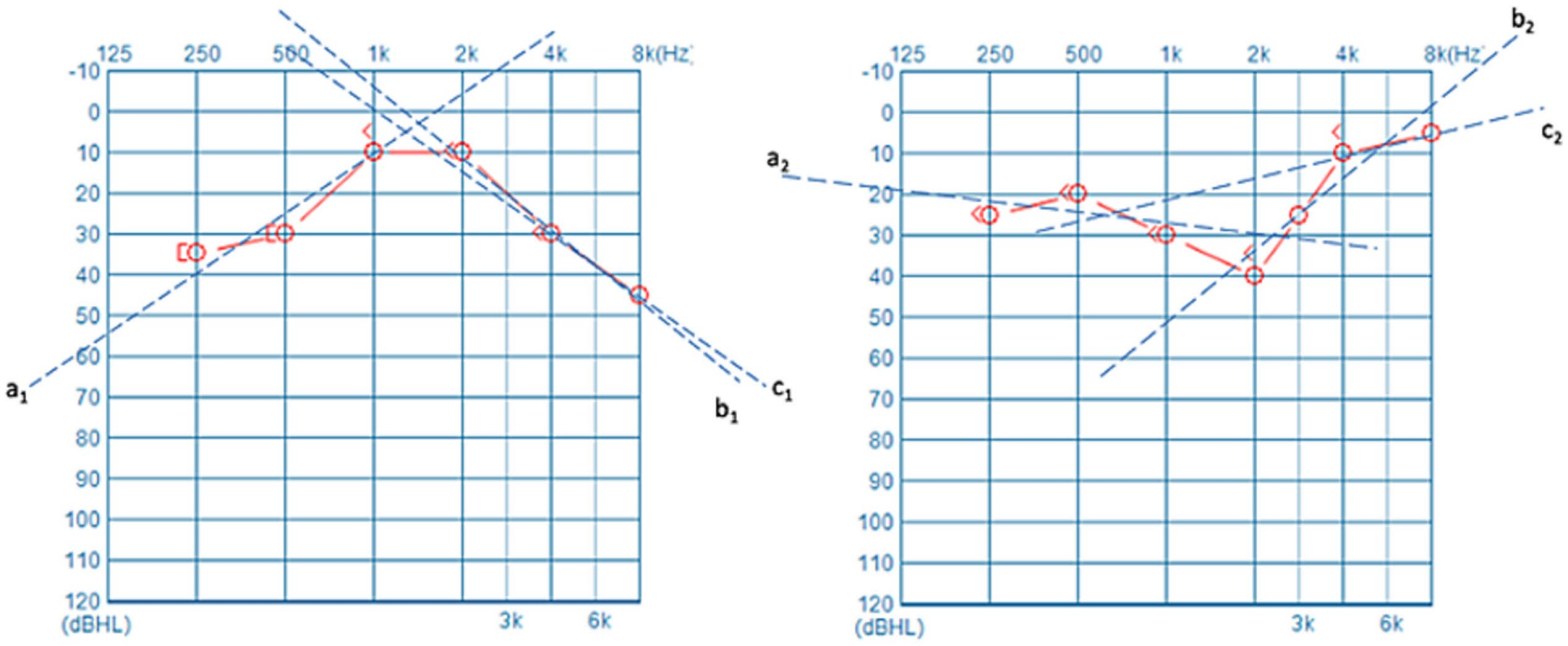
Three lines labelled as *a*, *b*, and *c* were employed to fit the PTA thresholds. These fitted lines succinctly and effectively represent the general trend of the PTA curve, with a particular emphasis on the high-frequency portion.

Figure 1 presents the audiograms of the unaffected ear of two patients with USSNHL. The mean hearing thresholds for both unaffected ears are approximately equivalent. However, the trajectories of their hearing threshold curves exhibit significant variability. This divergence may encapsulate unexplored latent information. To succinctly encapsulate the trends in hearing threshold curves, we devised three lines labelled *a*, *b*, and *c*, which are regression-fitted lines derived from specific frequency segments of the audiograms (Figure 2). Line *a* represents the regression line of the hearing threshold curve between 250 Hz and 1,000 Hz, while line *b* fits the curve between 2,000 Hz and 8,000 Hz. Moreover, recognizing the prevalence of a steep decline in hearing thresholds at higher frequencies among unaffected ears in clinical practice, we introduced line *c* as a regression fit for the hearing threshold curve between 4,000 Hz and 8,000 Hz, thus capturing this prominent feature. Subsequently, these three slopes, k*a*, k*b*, and k*c*, are extracted to represent an audiogram’s general trend. Employing Python 3.0, we then executed the K-means clustering algorithm to classify the extracted feature datasets from 229 patients with USSNHL based on their proximity. Clustering analysis was performed to partition these datasets into a finite number of clusters based on their similarity. For analytical convenience, we specified two clusters to be formed. Post-classification, patients within the same cluster exhibit closely aligned slopes k*a*, k*b*, and k*c*, indicating homogeneity in their unaffected ear audiogram characteristics; conversely, patients in different clusters display lower similarity in these characteristics. Having trained our model with the dataset from the 229 patients, we exported the trained clustering algorithm model (ds_cluster_model.pkl). We implemented a Python 3.0 script (predictor.py) that facilitates inputting audiogram data from an electronic spreadsheet (data for ds model.xlsx) to output cluster classifications. This resource maintains methodological transparency while adhering to computational efficiency principles through strategically optimized code architecture. You can find these documents in the appendices. This pipeline serves as a tool for broadly communicating and facilitating the validation of predictive measures for treatment outcomes in patients with USSNHL.

### 2.3 Compilation software and statistical methods

This study employs Jupyter Notebook 6.5.4 (Jupyter Team, https://jupyter.org) to execute the Python 3.0 programming language, facilitating the training of the cluster analysis model. All statistical computations were conducted using SPSS Statistics 29 (IBM, Armonk, NY, United States) and Prism 10 (Version 10.0.3, GraphPad Software, LLC). Continuous variables following a normal distribution are expressed as mean ± standard deviation, whereas those deviating from normality are represented by the median and interquartile range (IQR). For two groups of continuous variables that are typically distributed, the *p*-values were derived from Student’s *t*-tests. Non-normally distributed continuous variables were evaluated using the Mann–Whitney nonparametric test to determine p-values. Comparative analysis of categorical data relied on chi-square tests to determine p-values. Regression analysis encompassed univariate/multivariate linear regression and univariate/multivariate binary Logistics regression. Variables demonstrating *p* < 0.05 in univariate regression analysis were incorporated into multivariate regression models. Across all statistical comparisons, *p* < 0.05 was considered statistically significant. Statistical hypothesis testing was performed using a two-tailed approach with a 95% confidence interval (CI).

### 2.4 Ethical statement

This study was approved by the Ethics Committee of Beijing Tongren Hospital, CMU (TREC2024-KY057). It does not involve interventions or invasive procedures on human subjects.

## 3 Results

### 3.1 The clustering analysis model developed in this study

**Figure 3:**
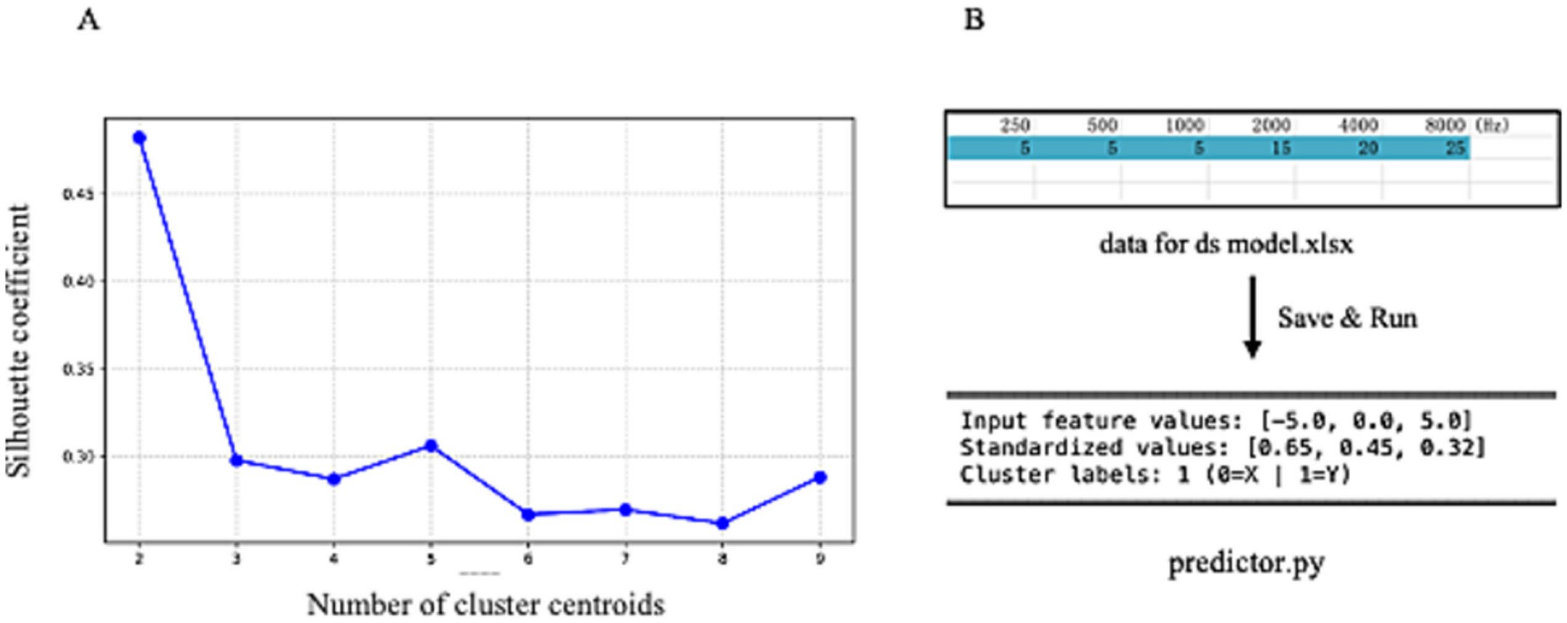
**(A)** Displays the silhouette coefficient plot for clustering analysis, with the x-axis representing the number of cluster centers and the y-axis corresponding to silhouette coefficients. The plot reveals the highest silhouette coefficient when the number of cluster centers is set to 2. **(B)** Illustrates the operational schematic of our clustering model. By entering audiometric data from unaffected ears into the provided digital interface, users can obtain definitive cluster assignments.

This study employed the hearing data from the unaffected ears of 229 patients suffering from USSNHL to train a clustering model and develop an input–output program. Figure 3A delineates the relationship between silhouette coefficients and cluster centroid numbers. The optimal silhouette coefficient of approximately 0.47 was attained when setting the cluster number to 2, demonstrating statistically validated clustering efficacy. This parameter configuration suggests bimodal distribution characteristics within the auditory threshold dataset. Figure 3B represents a schematic of our clustering results and the program’s operational flow. By inputting the PTA thresholds at the six frequencies of 250 Hz, 500 Hz, 1,000 Hz, 2,000 Hz, 4,000 Hz, and 8,000 Hz from the unaffected ear into the provided spreadsheet titled “data for ds model.xlsx” and saving it, the “predictor.py” can be executed using a compiler. The program automatically calculates and extracts the curve characteristics of the unaffected ear, normalizes the data, and then outputs the clustering results for the patient in the form of “0” or “1”, where “0” corresponds to Cluster X, and “1” corresponds to Cluster Y, as defined in this paper. This study ultimately provides the “Predict-Min” repository, containing minimal essential files and executable codes that enable clinical implementation of contralateral audiogram classification for novel patient cohorts. It is recommended that researchers download these materials for verification, ensuring all files are placed within the same directory and that the file names remain unchanged.

### 3.2 The clustering results and differences in treatment efficacy among 229 patients with USSNHL

**Figure 4:**
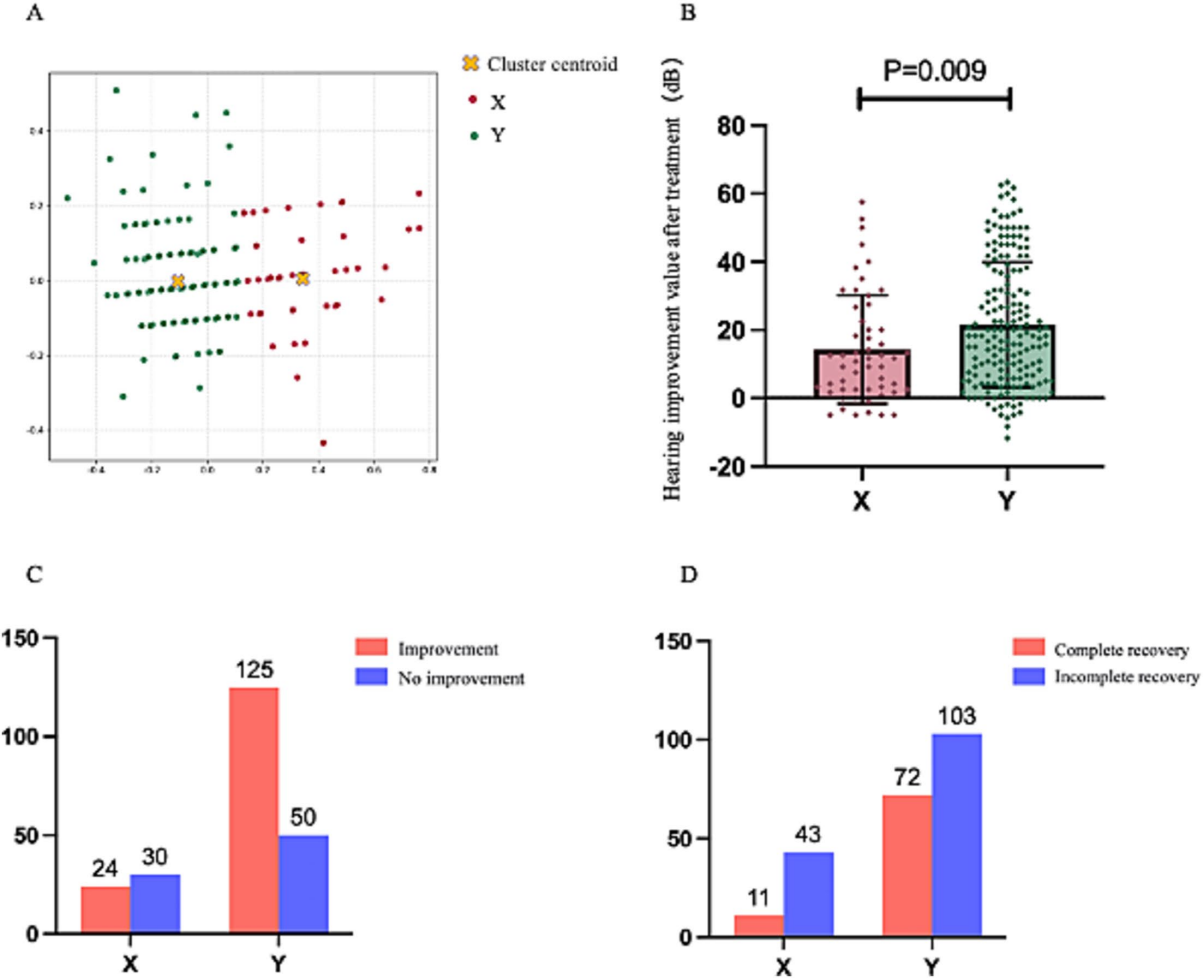
**(A)** Presents the post-clustering scatterplot of 229 patients, with the legend positioned in the upper-right corner. **(B)** Demonstrates the comparative analysis of post-treatment mean threshold improvement between the two clusters. **(C,D)** Illustrate statistical comparisons of treatment response rates and complete recovery rates across clusters, each annotated with case counts (numerical labels) and accompanied by legends in the upper-right quadrant.

A total of 229 patients were classified into two Clusters, X and Y, based on the audiometric characteristics of their unaffected ears using clustering algorithms. Ultimately, 54 patients were allocated to Cluster X. In comparison, 175 patients were assigned to Cluster Y, resulting in a patient ratio of approximately 1:3. Figure 4A depicts the cluster scatterplot, where X and Y coordinates represent cluster centroids surrounded by their corresponding data points.

Figures 4B–D present the treatment outcomes after the division of the 229 patients into two clusters. The mean improvement in post-treatment average hearing thresholds of patients in Cluster X was significantly lower than those in Cluster Y (14.27 ± 15.93 dB vs. 21.56 ± 8.32 dB, *p* = 0.009). Moreover, the cure rate of Cluster X patients was significantly lower than that of Cluster Y (*p* = 0.006), and the improvement rate was also significantly lower (*p* < 0.001). Consequently, regardless of the perspective considered, the prognosis of USSNHL patients categorized in Cluster X deteriorates significantly compared to those in Cluster Y. This corroborates the effectiveness of our clustering model as a robust tool for predicting prognosis.

### 3.3 Validation dataset construction using international diagnostic criteria

Table 1 presents a comparative analysis of the clinical data of the 229 patients, including 177 patients meeting international diagnostic criteria and the 52 patients excluded. Data of the age, the average hearing threshold of the affected ear after treatment, and the average hearing threshold of the unaffected ear do not conform to a normal distribution; therefore, in the table, the data are described using the minimum value, maximum value, median, and IQR. There is a statistically significant difference in age between the two groups of patients, with the age of the patient group that does not meet international diagnostic standards being overall younger (*p* < 0.001). However, despite the treatment, the average hearing threshold of the excluded patients’ affected ear remains significantly lower than that of the unaffected ear (*p* < 0.001), with a median difference of 6.70 dB between the two.

**Table 1:** Comparison of clinical data between the 177 included and the 52 excluded participants.

Characteristic	177 patients were included	52 patients were excluded	*p*
Age (years)			<0.0001
Minimum/maximum	18/82	21/57	
Median and IQR	49 (35, 63)	37.5 (32, 42.75)	
Gender (male/female)	87/90	18/34	0.064
Side (left/right)	89/88	34/18	0.039
Average hearing threshold of the affected ear after treatment (dB)			<0.0001
Minimum/maximum	1.67/120^a^	4.17/36.70	
Median and IQR	49.20 (27.50, 74.15)	16.70 (9.38, 21.28)	
Average hearing threshold of the unaffected ear (dB)			<0.0001
Minimum/maximum	0.83/87.50	1.67/20.83^b^	
Median and IQR	17.50 (10.83/28.34)	10.00 (8.54/15.00)	

a 120 dB indicates that even at the maximum sound level of the audiometric instrument, the sound remains inaudible. For calculation, it is set at 120 dB.

b There is a significant difference between the average hearing thresholds of the unaffected ear and the average hearing threshold of the affected ear post-treatment in the 52 excluded patients, with *p* < 0.001.

### 3.4 Validation results from 177 patients

**Figure 5:**
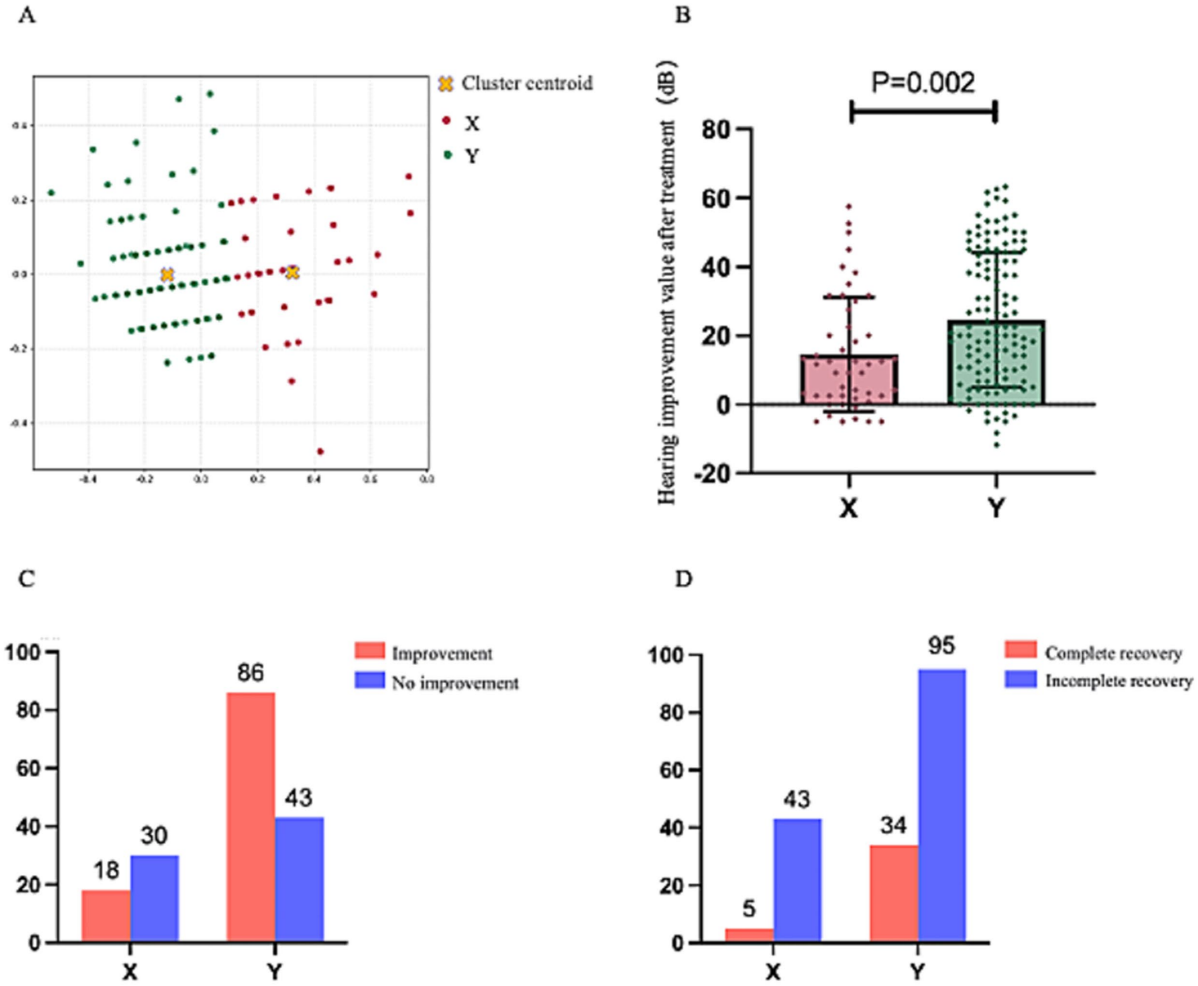
This figure maintains the analytical framework established in Figure 4. **(A)** Displays the post-clustering scatterplot of 177 patients, with the legend positioned in the upper-right quadrant. **(B)** Compares the post-treatment mean threshold improvement between the two clusters. **(C,D)** Present comparative analyses of treatment response rates and complete recovery rates, respectively, each accompanied by upper-right legends and numerical annotations indicating corresponding case counts.

After excluding 52 patients using international diagnostic criteria, we reapplied the model for clustering analysis to the remaining 177 patients. Among these 177 patients, 48 were clustered into Cluster X, while 129 patients were clustered into Cluster Y, maintaining a ratio of 1:3. Figure 5A depicts the cluster scatterplot, where X and Y coordinates represent cluster centroids surrounded by their corresponding data points. Figures 5B–D present the treatment outcomes after the division of the 177 patients into two clusters.

Like the aforementioned results, the improvement in average hearing thresholds post-treatment for patients in Cluster X was significantly lower than that for patients in Cluster Y (14.60 ± 16.63 dB vs. 24.56 ± 19.52 dB, *p* = 0.002). The cure rate for patients in Cluster X was significantly lower than that for Cluster Y (*p* = 0.0229), and the improvement rate was also significantly lower than that for Cluster Y (*p* < 0.001). These findings indicate that it still demonstrates favorable outcomes when our clustering model is applied to a population of USSNHL patients adhering to internationally recognized and more rigorous diagnostic criteria. These results substantiate the model’s broad applicability and potential for widespread utility and communication.

### 3.5 Linear regression and binary logistic regression analysis of the treatment outcomes for 177 patients

Following the preliminary confirmation of the correlation between our clustering results and treatment outcomes, to avoid confounding bias, we performed a regression analysis with treatment efficacy as the dependent variable, incorporating factors potentially influencing treatment effects among the 177 patients and their baseline data. It was undertaken to demonstrate that our clustering results indeed represent an independent factor rather than a misleading positive result attributable to confounding variables. Table 2 presents the results of univariate and multivariate linear regression analyses, utilizing the average improvement in hearing thresholds of the affected ear as the dependent variable.

**Table 2:** Results of univariate and multivariate linear regression for hearing improvements associated with affected ear.

Variables	Univariate					Multivariate				
	*β*	S. E.	*t*	*p*	*β* (95% CI)	*β*	S. E.	*t*	*p*	*β* (95% CI)
Cluster										
X					0.00 (Reference)					0.00 (Reference)
Y	9.96	3.18	3.14	0.002	9.96 (3.74–16.19)	6.52	3.29	1.98	0.049	6.52 (0.08–12.97)
Age (years)	−0.32	0.09	−3.58	<0.001	−0.32 (−0.50 to −0.14)	−0.26	0.09	−2.78	0.006	−0.26 (−0.44 to −0.08)
Audiogram type of the affected ear										
Upsloping					0.00 (Reference)					
Downsloping	−16.53	8.52	−1.94	0.054	−16.53 (−33.23 to 0.17)					
Flat	2.16	6.02	0.36	0.720	2.16 (−9.64 to 13.95)					
Profound	2.74	6.25	0.44	0.662	2.74 (−9.52 to 14.99)					
Side										
Left					0.00 (Reference)					
Right	−0.23	2.90	−0.08	0.938	−0.23 (−5.92 to 5.46)					
Gender										
Male					0.00 (Reference)					
Female	0.36	2.90	0.12	0.903	0.36 (−5.33 to 6.04)					

In Table 2, we utilized the average threshold improvement value of the affected ear post-treatment as the dependent variable. In contrast, the clustering results, age, audiogram type of the affected ear, gender, and side of the condition were employed as independent variables for both univariate and multivariate linear regression analyses. Univariate regression analysis was implemented to explore the correlation between variables, while multivariate analysis was conducted to mitigate confounding factors. The variable marked as “Reference” in the table is a reference variable. According to the results of the multivariate analysis, it is evident that the average threshold improvement value is significantly correlated with the clustering results and age. Specifically, patients categorized into Cluster Y exhibited a 6.52 dB higher average threshold improvement compared to those in Cluster X (*p* = 0.049). Furthermore, for each additional year of age, the average threshold improvement value decreased by 0.26 dB (*p* = 0.006). No linear relationship was observed in the linear regression analysis between the audiogram type of the affected ear, the side of the condition, and gender about the average threshold improvement value.

Table 3 sets the improvement status as a binary dependent variable, with “Improvement” coded as “0” and “No improvement” coded as “1.” The independent variables include clustering results, quartile classification of age, audiogram type, side of the condition, and gender. The variable labelled “Reference” serves as the intra-group reference value. This table categorizes age into four classes based on quartiles, with the youngest 25% of patients designated as “1.” Conversely, the eldest 25% are labelled as “4.” The boundary values for quartile classification are specified in Table 1, where “1” represents an age range of 18–35 years, “2” represents 36–49 years, “3” represents 50–63 years, and “4” represents 64–82 years. From this table, we can conclude that the probability of “No improvement” for patients in Cluster Y is approximately half that of patients in Cluster X (OR = 0.43, *p* = 0.029). The likelihood of “No improvement” in the age group of 64–82 years is about three times that of the age group of 18–35 years (OR = 3.27, *p* = 0.019). Patients with “downsloping” or “flat” types exhibit a significantly higher risk of “No improvement” compared to those with “upsloping” types, with OR values of 32.27 (*p* = 0.009) and 9.25 (*p* = 0.043), respectively. Similarly, under a particular statistical error margin, the risk of “No improvement” in patients with “profound” type is also markedly elevated (OR = 7.98, *p* = 0.063). Additionally, no side or gender impact on improvement status was observed in the binary Logistic regression analysis. In addition, we conducted univariate and multivariate binary Logistic regression analyses with complete recovery status as the dependent variable. Patients who achieved “Complete recovery” were coded as “0,” while the remaining patients were coded as “1.” The results of the univariate regression were like those mentioned above; however, the multivariate regression results showed no significant correlation, which may be attributable to insufficient sample size.

**Table 3:** Results of univariate and multivariate binary logistic regression analysis on the improvement status of treatment for affected ear.

Variables	Univariate					Multivariate				
	*β*	S. E.	*Z*	*p*	OR (95% CI)	*β*	S. E.	*Z*	*p*	OR (95% CI)
Cluster										
X					1.00 (Reference)					1.00 (Reference)
Y	−1.20	0.35	−3.42	<0.001	0.30 (0.15–0.60)	−0.84	0.39	−2.18	0.029	0.43 (0.20–0.92)
Age (IQR)										
1 (18–35)					1.00 (Reference)					1.00 (Reference)
2 (36–49)	−0.03	0.48	−0.07	0.945	0.97 (0.37–2.50)	−0.23	0.50	−0.46	0.646	0.79 (0.30–2.13)
3 (50–63)	0.65	0.46	1.40	0.161	1.91 (0.77–4.71)	0.45	0.49	0.93	0.355	1.57 (0.60–4.08)
4 (64–82)	1.40	0.47	2.96	0.003	4.04 (1.60–10.19)	1.18	0.51	2.34	0.019	3.27 (1.21–8.82)
Audiogram type of the affected ear										
Upsloping					1.00 (Reference)					1.00 (Reference)
Downsloping	3.00	1.26	2.37	0.018	20.00 (1.68–238.55)	3.47	1.33	2.62	0.009	32.27 (2.39–435.65)
Flat	2.00	1.07	1.88	0.061	7.41 (0.91–60.10)	2.23	1.10	2.02	0.043	9.25 (1.07–80.18)
Profound	1.94	1.08	1.79	0.073	6.97 (0.83–58.24)	2.08	1.12	1.86	0.063	7.98 (0.90–71.14)
Side										
Left					1.00 (Reference)					
Right	−0.21	0.31	−0.70	0.484	0.81 (0.44–1.47)					
Gender										
Male					1.00 (Reference)					
Female	0.08	0.31	0.27	0.788	1.09 (0.60–1.98)					

### 3.6 Disparities in characteristic data between two patient clusters

**Figure 6:**
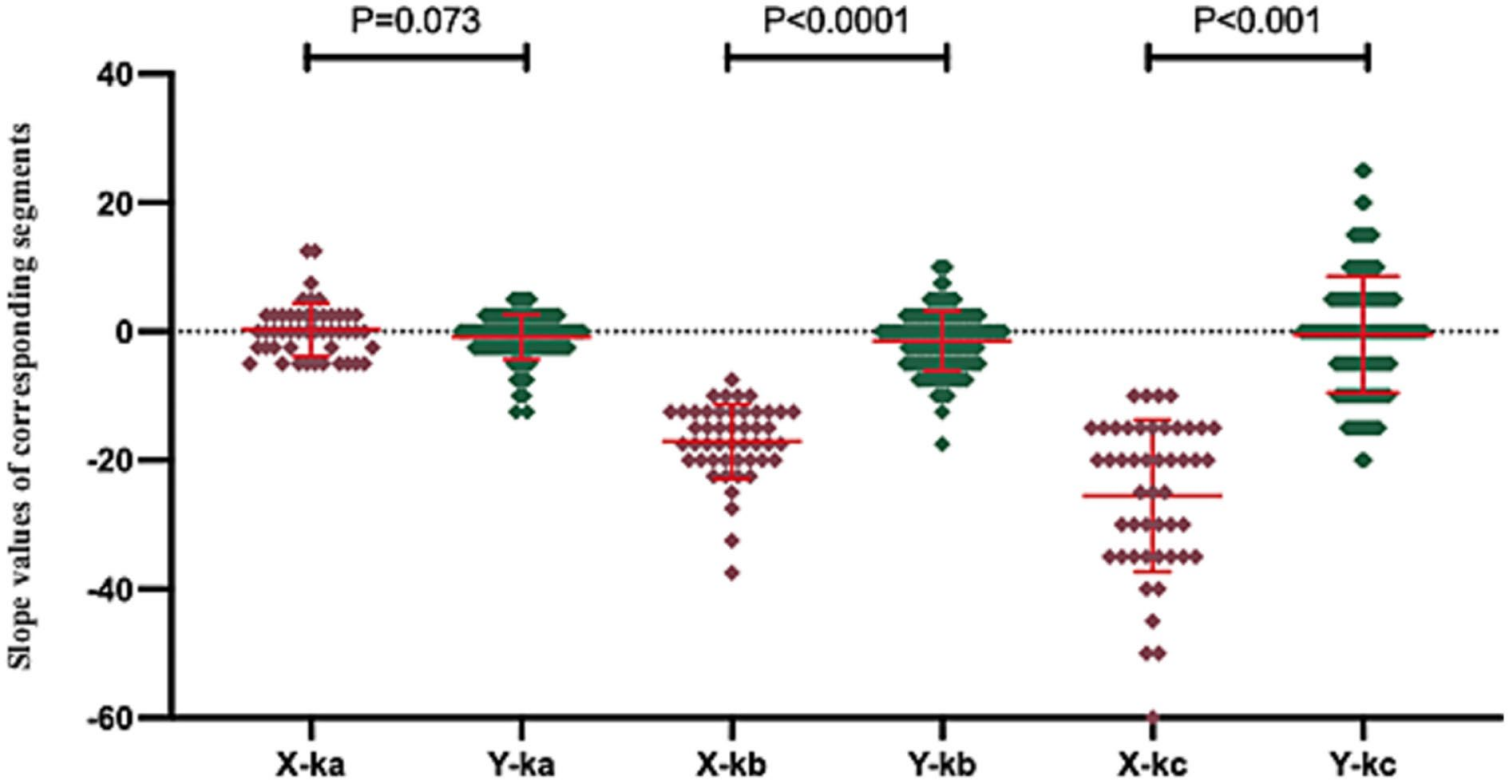
This figure illustrates the distribution of characteristic data values across the two patient clusters. In this figure, “X-k*a*” denotes the slope of fitted linear regression line a for Cluster X, with analogous labeling applied to other parameters. These slope parameters were standardized such that values greater than 0 reflect an upward trend in the corresponding curve segment, values less than 0 indicate a downward trend, and values equal to 0 represent a flat pattern, where larger absolute magnitudes correspond to steeper directional changes.

Given the inherent “black-box” nature of cluster analysis—which processes input data to generate categorical classifications without explicitly revealing the substantive distinctions between clusters, thereby complicating the interpretation of inter-cluster differences—we conducted a comprehensive statistical aggregation of three transformed slope parameters (k*a*, k*b*, and k*c*) across the 177 clustered patients, as depicted in Figure 6. Comparative analysis revealed marginal significance between X-k*a* and Y-k*a* (*p* = 0.073), while X-k*b* versus Y-k*b* and X-k*c* versus Y-k*c* both exhibited high significance (*p* < 0.001). As illustrated in Figure 6, Cluster Y demonstrated mean slope values for k*a*, k*b*, and k*c* approximating zero, indicative of flatter overall morphologies in contralateral audiometric curves. In contrast, Cluster X displayed pronounced descending trends within the 2,000–8,000 Hz and/or 4,000–8,000 Hz frequency ranges. This evidence collectively suggests that the predominant discriminative factor underlying the bipartite clustering of the 177 patients resides in mid-to-high frequency descent patterns within the contralateral ear, with low-frequency curve morphology exerting a secondary, non-definitive influence on the classification outcome.

## 4 Discussion

### 4.1 Findings of the study

In this study, we used Python 3.0 to extract audiometric data from the unaffected ear of 229 patients with USSNHL who met the diagnostic criteria established in China. We achieved effective clustering results and ultimately trained a binary clustering model, demonstrating the capability to output clustering results upon inputting audiometric data from the unaffected ear. This feature facilitates the broad communication of our findings and allows for public verification. Following the initial results obtained via the chi-square test, we performed a regression analysis to derive richer and more rigorous outcomes. Thus, we can assert that our clustering analysis results represent independent prognostic factors for patients with USSNHL after controlling for confounding biases. Moreover, our findings affirm that age and the audiogram type of the affected ear are also independent factors influencing the prognosis of USSNHL, which aligns with the findings of previous researchers (11). Additionally, we have provided corresponding quantifiable metrics.

The average improvement in hearing threshold following treatment for patients in Cluster Y is merely 6.52 dB higher than that for patients in Cluster X in the multivariable linear regression analysis, but this difference holds clinical significance. The human auditory system demonstrates a differential threshold for sound intensity discrimination substantially lower than 6 dB, suggesting a theoretical capacity to detect 6 dB differences. Nevertheless, this magnitude of hearing threshold shift might not manifest clinically significant quality-of-life impairment (12). The prognosis of USSNHL often depends on multiple factors. For example, when combined with age, the predictive value of cluster analysis may be significantly enhanced, leading to dramatically different implications for the patient. However, from an energy perspective, a hearing loss exceeding 6.52 decibels (dB) indicates that the auditory system’s ability to perceive sound stimuli is reduced to one-fourth of its original capacity (approximately a 75% decrease in energy sensitivity).

### 4.2 Hypotheses to explain the findings

We propose the following two hypotheses regarding the close correlation between our clustering results and therapeutic outcomes. First, we observed that upon completion of clustering, patients in Cluster X demonstrated audiometric profiles of their unaffected ears that more closely resembled the characteristics of Patient (A) depicted in Figure 1. The trend indicates that auditory levels deteriorate as the frequency approaches the extreme boundaries, particularly in the high-frequency range, where this phenomenon is especially pronounced. This trend may signal a substantial degree of cochlear dysfunction within the 40% range not reflected in the audiograms of these individuals. Consequently, it is comprehensible that such patients exhibit poor recovery outcomes following exposure to etiology associated with SSNHL. We have not retrieved any previous publication that substantiates this hypothesis, which indicates that this research constitutes a novel exploration. Second, although the feature data we extracted only encompasses the slope information of the three fitted lines, this slope data inherently contains some information pertaining to the contralateral auditory threshold. For example, if an individual displays a steep decline in the mid-to-high frequency region, the slopes of their lines *b* and *c* will undoubtedly increase. At the same time, concurrently, their average hearing threshold values would rise. Despite the absence of collected average hearing threshold data for patients’ unaffected ears, for the sake of rigor, such information may have been inadvertently incorporated into the clustering model. Therefore, the hearing threshold levels of this unaffected ear could be associated with the functional status of the cochlea prior to onset, thereby enhancing the validity of our clustering model (13).

### 4.3 Significance of cluster analysis in the study

The value of the clustering analysis model in this study lies in its provision of a novel standard for classifying individuals with USSNHL. Instead of offering a dichotomous cutoff value that arbitrarily divides the population into two categories, it considers the relationships among various features and data within a more comprehensive dataset. Although these features and relationships cannot be precisely delineated, they can be effectively leveraged. We provide a spreadsheet and a Python program to ensure this research can be validated and utilized.

### 4.4 Diagnostic criteria for SSNHL

In this study, we also present partial clinical data for 52 excluded patients who met the diagnostic criteria outlined in the “Chinese guidelines” but did not conform to the internationally recognized diagnostic criteria. Despite receiving aggressive treatment, the hearing in the affected ear deteriorated by 6.70 dB compared to the unaffected ear. It indicates that even after treatment, this subset of individuals exhibits statistically significant differences in hearing compared to before the onset of the condition, although a difference of 6.70 dB may not affect the quality of life. Nevertheless, this group would not even be diagnosed with a condition under international diagnostic criteria. The question of whether these patients should receive treatment and to what extent, indeed, warrants further discussion. Confusion about diagnostic criteria and treatment options is not only present in China (14, 15).

### 4.5 Limitations and future directions

The methodological design of this study is highly innovative and provides a new way of categorizing audiological data by means of cluster analysis. However, an inherent problem with this type of research is the “black box” nature of cluster analysis. We can only observe the input data and the output results. It is difficult to describe in detail what the algorithm does with the data. There is also no value in trying to find a clear distinction between the two clusters of patients, as simply using one of the hearing data as a cutoff value to categorize the patients in this study does not yield positive results. We were only able to obtain an approximate impression of the two clusters of patients, which is an inherent limitation of the methodology. However, this method is still valuable for generalization because the results of cluster analysis can provide a new statistical basis for the classification of hearing data. This study specifically enrolled patients presenting with USSNHL based on their chief complaint during the current medical encounter, with explicit exclusion of those having prior contralateral SSNHL episodes through medical history review. However, potential inclusion of patients with sequential bilateral SSNHL cannot be entirely ruled out due to possible inaccuracies in symptom perception and self-reporting—specifically, instances where initial mild symptoms in the first affected ear might have escaped clinical attention. Nevertheless, given the low epidemiological prevalence of sequential bilateral SSNHL [approximately 2% of SSNHL cases, predominantly simultaneous bilateral onset with minimal sequential occurrences (16)] coupled with our study’s substantial sample size, this potential bias does not substantially compromise the validity of our findings.

In subsequent research, we can apply this clustering model to analyze healthy individuals’ audiograms under age balance, observing the proportion of Cluster X and Cluster Y. In individuals with USSNHL, this ratio is 1:3; if a significant change in this proportion is observed in healthy individuals, it suggests that this audiometric feature not only indicates the prognosis of SSNHL patients but correlates with the incidence of the condition. Should the findings be validated, subsequent investigations could focus on determining whether this subpopulation exhibits genetic predispositions—such as alterations in human leukocyte antigen (HLA) alleles—that may contribute to elevated disease susceptibility (17). This dual-axis approach would enable comprehensive understanding of patient vulnerability through integrated genetic and audiometric perspectives. Additionally, we can explore whether there are differences in specific candidate endolymphatic pathological biomarkers [such as prestin (18, 19), otolin-1 (20), cochin (21)] between these two clusters, thereby substantiating potential pathologic states in the cochlea.

## 5 Conclusion

This study demonstrates that our clustering model effectively categorizes individuals with USSNHL into two distinct clusters. The resulting clusters serve as a reliable, accessible, and independent prognostic indicator for USSNHL. Notably, the model’s effectiveness remains robust across different diagnostic criteria. The model’s predictive power may stem from its ability to extract latent auditory information from the unaffected ear, enabling binary classification of cochlear functional states. The data exerting the most significant influence on clustering analysis outcomes were derived from the evolving auditory threshold patterns in the posterior segment of audiometric curves obtained from unaffected ears. This observation indicates a strong correlation between mid-to-high frequency threshold progression in the contralateral ear and clinical prognosis among patients with USSNHL.

## Data Availability

The raw data supporting the conclusions of this article will be made available by the authors, without undue reservation.
