# Supplementary figures and images for: Predicting the prognosis of patients with sudden sensorineural hearing loss by analyzing the audiometric curve of the unaffected ear
Source: Front Neurol. 2025 May 30;16:1575122. doi: 10.3389/fneur.2025.1575122 (PMC12162312; doi:10.3389/fneur.2025.1575122)

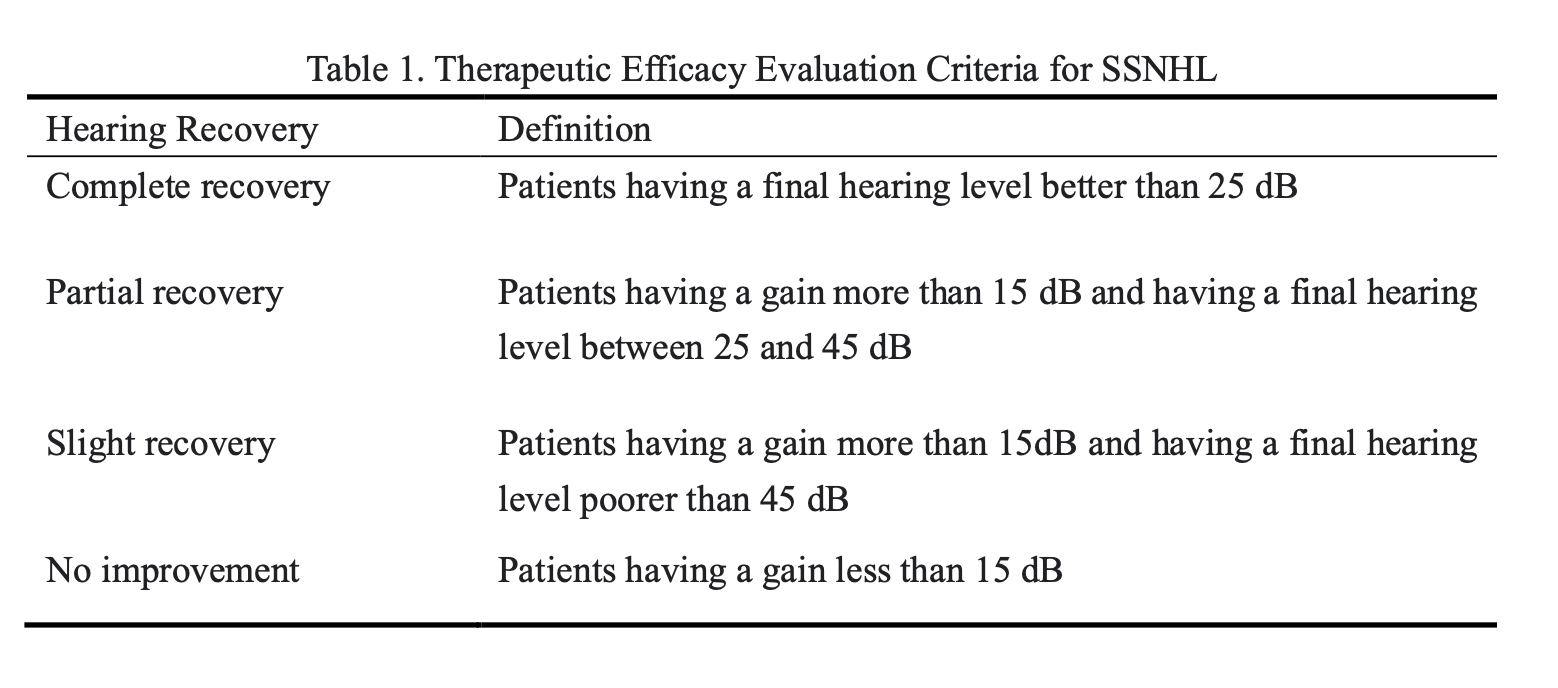

Supplement: Supplementary file 1 [file Data_Sheet_1.zip › Supplemental Data File/Therapeutic Efficacy Evaluation Criteria.png]
